# Design, Simulation, and Fabrication of a New Three-Axis Inertial Switch with a Triangular Movable Electrode Structure

**DOI:** 10.3390/mi14010094

**Published:** 2022-12-30

**Authors:** Wenguo Chen, Rui Wang, Huiying Wang, Zhen Yang

**Affiliations:** 1 Key Laboratory of Advanced Energy Materials Science and Technology, College of Information Engineering, Qujing Normal University, Qujing 655000, China; 2 School of Electronic and Information Engineering/School of Integrated Circuits, Guangxi Normal University, Guilin 541004, China

**Keywords:** three-axis inertial switch, movable electrode structure, dynamic response performance, triangular structure, threshold

## Abstract

A new three-axis inertial switch is proposed. The triangle-structured movable electrode is designed to improve the inertial switch’s dynamic response performance, especially the movable electrode’s dynamic stability performance. The static mechanical analysis indicated that the displacement of the movable electrode to the fixed electrode in the sensitive direction is the minimum when the acceleration is applied to this designed inertial switch. The dynamic simulation analysis showed that the threshold of the designed inertial is about 235 g. The threshold in the non-sensitive direction is about 240 g, 270 g, 300 g, and 350 g when the directions of applied acceleration deviate 15°, 30°, 45°, and 60° from the sensitive direction, respectively. These results indicated that the designed inertial could resist the impact in non-sensitive directions and improve the stability in sensitive directions. The prototype of the inertial switch was fabricated and tested successfully. The testing results indicate that the threshold of the fabricated inertial switch is about 219 g. The test results verify the dynamic stability performance of the designed inertial switch.

## 1. Introduction

As a passive sensor, the inertial switch is widely used in remote monitoring areas with limited power supply due to its standby power consumption being zero in the untriggered state [1,2,3]. As a “near zero power” sensor, it can also be used to monitor collisions that occur during the transport of particular goods [4,5,6,7]. Over the years, with the development of intelligent control systems, the inertial switch may be used as an intelligent inertial switch in a vibration system [8]. For example, when the inertial switch is installed on the ground to count the number of vehicles passing, the test system will often be subjected to inertial impact from different directions, but the system is required to only perceive the inertial impact from the vertical direction of the ground or the characteristic angle with the ground, so as to ensure the effective count of the number of vehicles passing. Therefore, improving the dynamic response performance of inertial switches is urgent. The excellent dynamic response performances of the inertial switch include contact stability and high single-axial sensitivity. The elimination of contact bounce manifests in good contact stability, and high directional sensitivity can effectively avoid the false trigger caused by the impact from the non-sensitive direction.

Compared with the piezoelectric microswitch [9] and electrostatic microswitch [10], the dynamic response performance of the piezoelectric microswitch and electrostatic microswitch is more stable and the closing time of the switch is controllable. The inertial switch proposed in this paper realizes the switch closure under the drive of inertial force. Compared with piezoelectric and electrostatic microswitches, the biggest advantage of inertial microswitches is that they do not need power supplies when they are not working. However, dynamic response performance and contact time are the main factors affecting device reliability. Therefore, in order to improve the dynamic response performance, a triangle movable electrode structure is proposed. Compared with our previous work [11], the stability of the triangle movable electrode structure is higher than that of the square structure. At the same time, based on our previous research, the elastic movable electrode structure can effectively realize the elastic contact and prolong the contact time.

In the structural design of MEMS inertial switch, the suspended structure composed of a spring and mass block is often used as the inertial impact sensitive structure. This multi-DOF elastic suspension system has the characteristics of being multi-modal, having a broad spectrum, and having increasing response frequency with increasing design threshold [12,13,14,15]. This suspension system is composed of a spring and mass with multi-degrees suitable for most inertial switching applications. The wide frequency response characteristics make this structure responsive in most inertial impact monitoring systems. However, for some inertial monitoring environments where the orientation and threshold are very accurate, this characteristic may lead to the false triggering of non-sensitive directional shocks. In order to improve the contact stability and direction sensitivity of the inertial switch, a triangular structure design of the inertial switch is proposed in this paper, and the experimental prototype is successfully fabricated. The theoretical analysis and experimental test results show that the contact stability and direction sensitivity of the proposed three-axis sensitive inertial switch is excellent.

## 2. Structure Design

**Figure 1:**
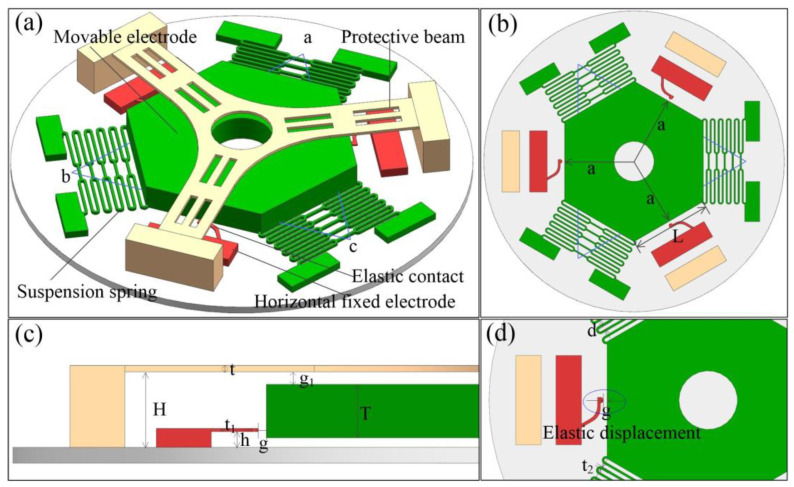
The structure of the designed three-axis inertial switch. (**a**) the 3D structure diagram of the inertial switch; (**b**) the top view of the structure; (**c**) one half of a lateral view of the structure; (**d**) the elastic fixed electrode.

The structure of the designed three-axis inertial switch is shown in Figure 1a. The main structure is composed of three parts. The hexagonal mass block connected to three sets of snake springs is designed as a movable electrode; the other ends of the springs are connected to the fixed block. The three fixed ends of the movable electrode form a triangular layout in three-dimensional space. Three fixed elastic electrodes are distributed on the edges of the hexagonal mass block, forming three sensitive directions that can be triggered. The structure with three fixed ends and another three movable sides determined three accurate, sensitive directions attributed to the triangle’s stability. The cross cantilever above the mass block can effectively restrain the bounce of the mass block in the Z direction. Figure 1b is the top view of the inertial switch, which shows the three sensitive directions. Figure 1c is the half of a side view of the structure. Figure 1d shows the elastic displacement between the mass block and fixed electrode. The main structural parameters of the designed inertial switch are shown in Table 1.

## 3. Theoretical Analysis and Simulation

### 3.1. Static Mechanical Analysis

**Figure 2:**
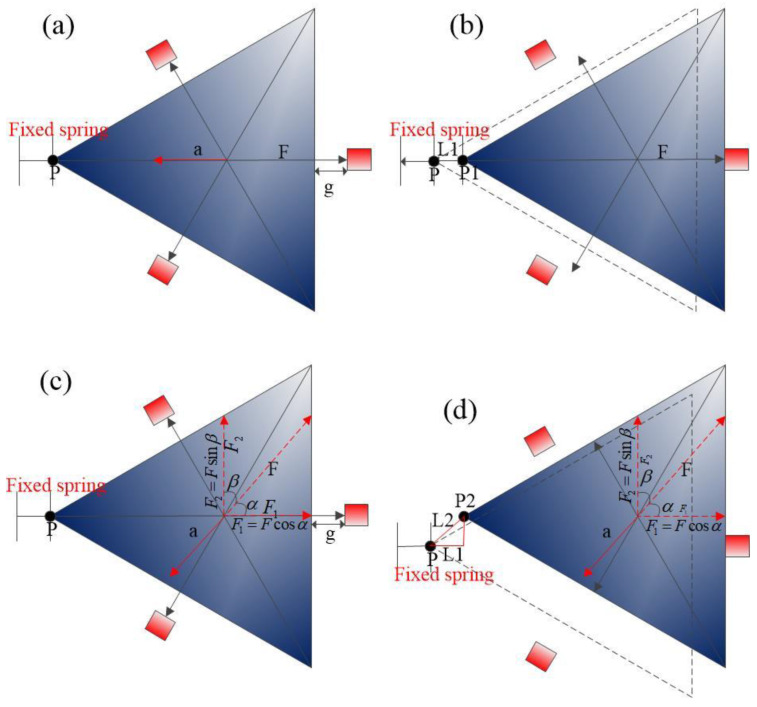
(**a**) The schematic diagram of a triangular movable electrode acting in a sensitive direction; (**b**) the movable electrode contacts with the fixed electrode in a sensitive direction; (**c**) the schematic diagram of a triangular movable electrode acting in a non-sensitive direction; (**d**) the movable electrode contacts with the fixed electrode in a non-sensitive direction.

The schematic diagram of the contact process of the designed three-axis inertial switch is shown in Figure 2. According to F=−ma, when the inertial switch is shocked in a sensitive direction, the proof mass moves towards the fixed electrode, which is attributed to the action of force F, as shown in Figure 2a. Furthermore, the direction of the force is opposite to the direction of the acceleration. Then, one end of the proof mass moves from point P to P1; the spring is stretched L1, and L1 = g, as shown in Figure 2b. When the inertial switch is shocked in the direction of deviation from the sensitive direction α, as shown in Figure 2c, the angle between the direction of the force and the sensitive direction is α, and the proof mass moves towards the fixed electrode attributed to the action of force F_1_. According to the vector decomposition method, one can obtain $F_{1}=F\cos\alpha$, $F_{2}=F\sin\beta$. The proof mass moves towards to fixed electrode in a sensitive direction if the angle α<β<90°, the movable electrode contact with the fixed electrode attributed to the force of F_1_, as shown in Figure 2d. At this moment, one end of the movable electrode moves from point P to point P2, and the spring elongation is L2. As can be seen from Figure 2d, the length of L1 is equal to the gap between the movable electrode and the fixed electrode, and the length of L2 is far greater than L1.

**Figure 3:**
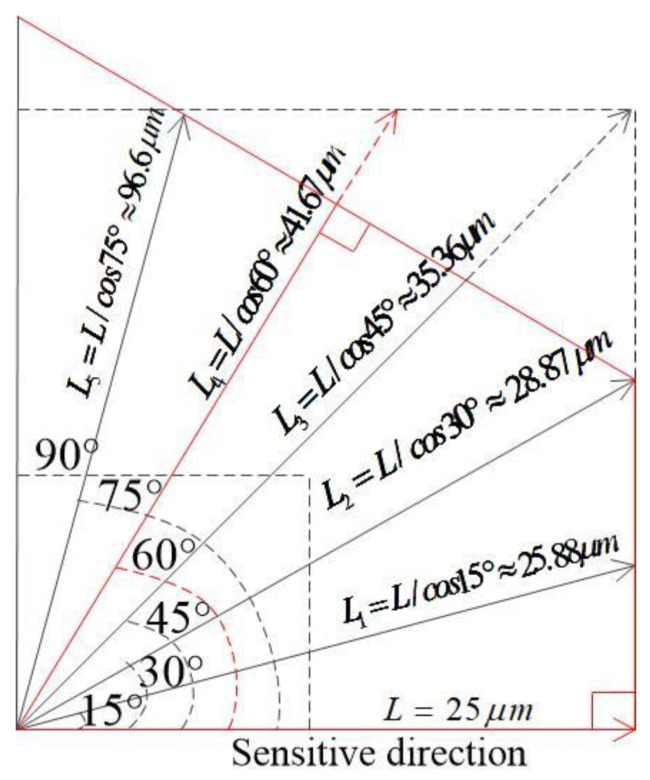
The schematic diagram of the displacement of proof mass when the direction of impact acceleration deviates from the sensitive direction of the inertial switch.

According to the law of trigonometric row functions, we can obtain $L_{n}=L/cos\theta$. When the direction of impact acceleration deviates from the sensitive direction of the inertial switch by 15°, 30°, 45°, 60°, and 75°, the displacement of the movable electrode is 25.88 μm, 28.87 μm, 35.36 μm, 41.67 μm, 96.6 μm, respectively. Because in the hexagonal structure, the angle cycle is 60°, in this design, the maximum deviation of the angle is 60°. The schematic diagram of the displacement of the proof mass is shown in Figure 3.

The following conclusion can be drawn from the above analysis: when the movable electrode with a triangular structure is shocked in the non-sensitive direction, and the greater the deviation of the acceleration direction from the sensitive direction, the greater the threshold acceleration. Thus, the threshold gap between the sensitive and non-sensitive direction is effectively ensured, the inter-axis interference and the probability of false trigger are reduced, and the stability of the device is improved.

### 3.2. Dynamic Simulation Analysis

**Figure 4:**
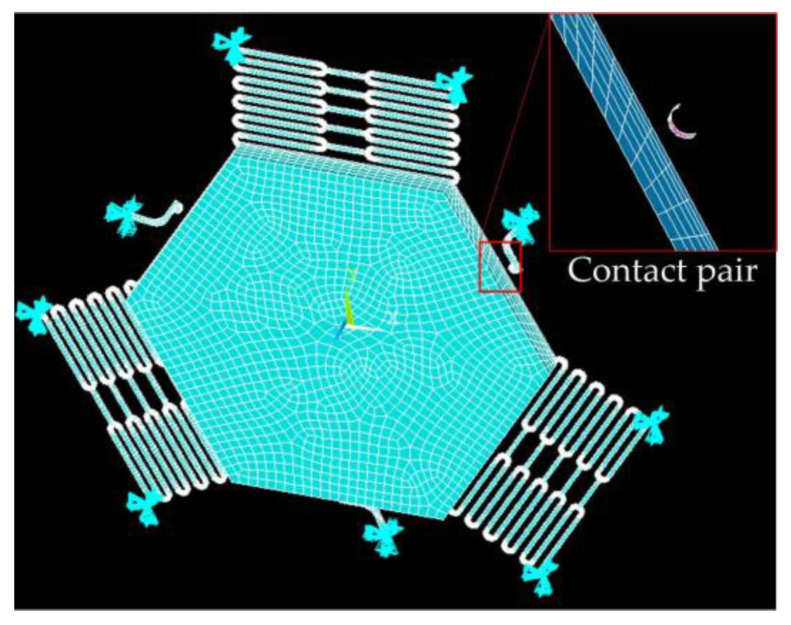
The FE model of the designed three-axis inertial switch.

**Figure 5:**
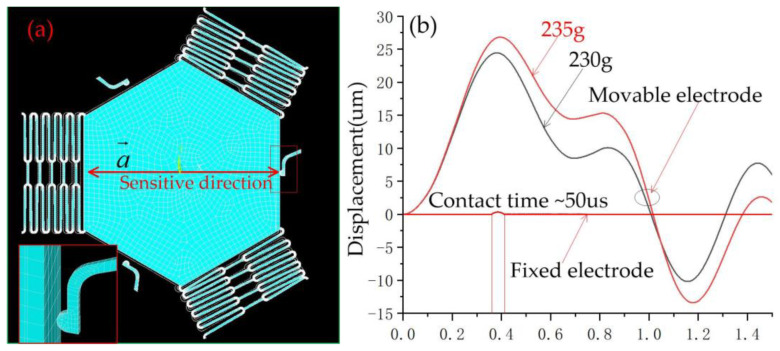
(**a**) Schematic diagram of movable electrode in contact with fixed electrode in a sensitive direction; (**b**) the dynamic contact process curves of movable and fixed electrodes in a sensitive direction.

The dynamic contact process was simulated by ANSYS finite element analysis software, and the finite model (FE) is shown in Figure 4. The model’s structure parameters are shown in Table 1. The ends of the springs and fixed elastic electrodes wee constrained to zero degrees of freedom. The nickel (Ni) electroplated by surface micromachining technology was selected as the structure, the material parameters of Young’s modulus and density were chosen to be 165 GPa and 8.96 g·cm^−3^, respectively [16]. To investigate the threshold of the designed three-axis inertial switch, the 230 g and 235 g accelerations with a pulse width of 1 ms were applied to the FE model in a sensitive direction, as shown in Figure 5a; the dynamic response curves of proof mass are shown in Figure 5b, which indicates that the displacement of proof mass decreases with the decrease of the peak acceleration. The displacement is about 26 μm when the acceleration is 235 g and the movable electrode contacts the fixed electrode, as shown in Figure 5a. Therefore, 235 g can be judged as the threshold acceleration. The dynamic contact process was obtained, which indicates that the response time is about 3.5 ms and the contact time is about 50 μs, as shown in Figure 5b.

In order to evaluate the stability of the triangular-structure inertial switch when the accelerations are applied to non-sensitive directions, the acceleration with different peaks was applied to the inertial switch in the directions away from the sensitive directions of 15°, 30°, 45°, and 60°, respectively.

**Figure 6:**
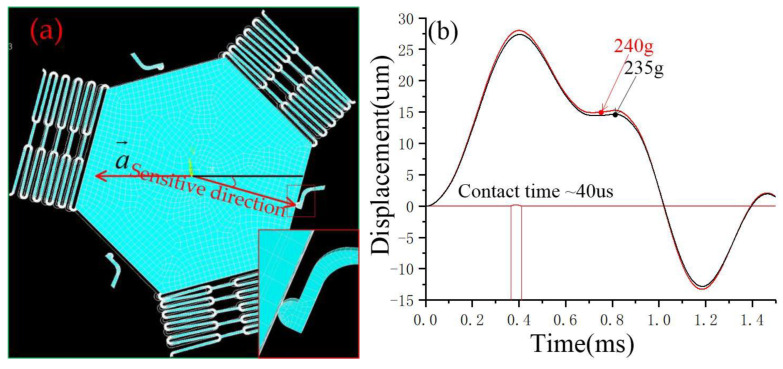
(**a**) Schematic diagram of a movable electrode in contact with a fixed electrode in the directions away from the sensitive direction of 15°; (**b**) the dynamic contact process curves of movable and fixed electrodes in the directions away from the sensitive direction of 15°.

Figure 6a shows the acceleration applied to the movable electrode in the direction of 15°. The accelerations with the peak value of 235 g, and 240 g were applied to the inertial switch in the directions away from the sensitive direction of 15°, respectively. The dynamic response curves shown in Figure 6a indicate that the movable electrode contacts with fixed electrodes when the acceleration is about 240 g, and the contact time is about 40 μs.

**Figure 7:**
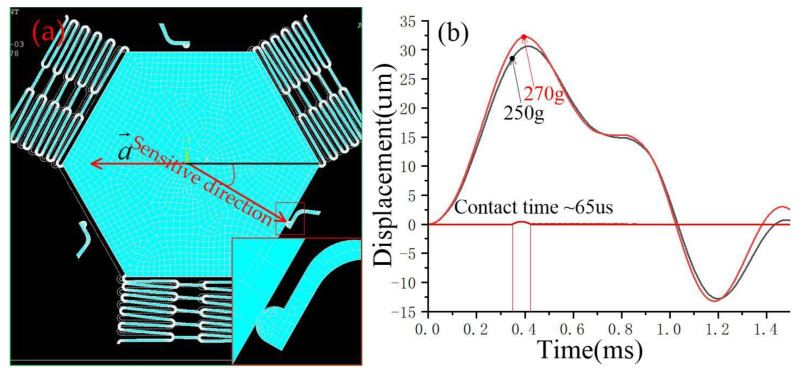
(**a**) Schematic diagram of a movable electrode in contact with a fixed electrode in the directions away from the sensitive direction of 30°; (**b**) the dynamic contact process curves of movable and fixed electrodes in the directions away from the sensitive direction of 30°.

**Figure 8:**
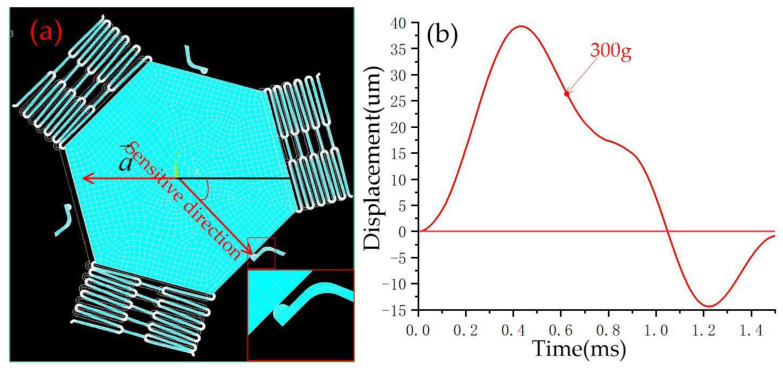
(**a**) Schematic diagram of a movable electrode in contact with a fixed electrode in the directions away from the sensitive direction of 45°, (**b**) the dynamic contact process curves of movable and fixed electrodes in the directions away from the sensitive direction of 45°.

**Figure 9:**
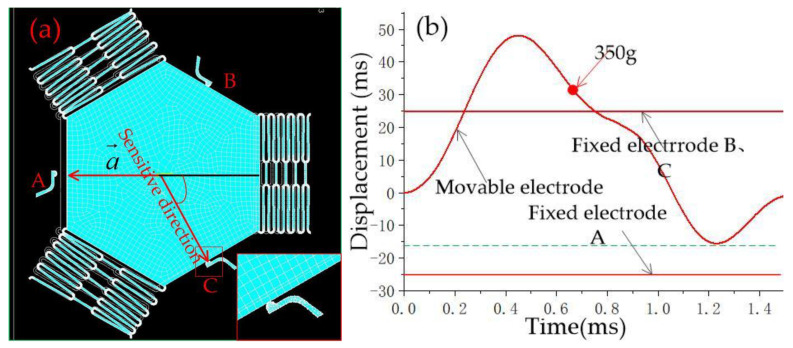
(**a**) Schematic diagram of a movable electrode in contact with a fixed electrode in the directions away from the sensitive direction of 60°, (**b**) the dynamic contact process curves of movable and fixed electrodes in the directions away from the sensitive direction of 60°.

When the acceleration direction deviates from the sensitive direction by 30°, as shown in Figure 7a, the simulation results show that the movable electrode and the fixed electrode realize contact at 270 g, and the contact time is about 65 μs, as shown in Figure 7b. Figure 8 and Figure 9 show that the thresholds of the designed inertial switch are 300 and 350 degrees at 45° and 60°, respectively. Figure 8a is the schematic diagram of the movable electrode in contact with the fixed electrode in the directions away from the sensitive direction of 45°; Figure 8b is the dynamic contact process curves of the movable and fixed electrode in the directions away from the sensitive direction of 45°. Figure 9a is the schematic diagram of the movable electrode in contact with the fixed electrode in the directions away from the sensitive direction of 60°, Figure 9b is the dynamic contact process curves of the movable and fixed electrode in the direction away from the sensitive direction of 60°. The dynamic response curves indicated that there is no elastic deformation when the acceleration is about 300 g or 350 g. This is because when the acceleration direction deviates from the sensitive direction, the direction of force will deviate from the deformation direction of the fixed electrode. The friction force is ignored in the simulation, resulting in a small force of the fixed electrode, so there is no elastic deformation.

Figure 9 shows that when 350 g acceleration was applied to the direction opposite to the sensitive direction (direction A), the movable electrode contacted the fixed electrodes B, and C, as shown in Figure 9a, but the fixed electrode did not show elastic deformation, and the contact time did not extend, as shown in Figure 9b. It also shows that when overload acceleration is applied in the opposite direction of the sensitive direction, the flexible electrode with elastic recovery will not be in contact with the fixed electrode in the sensitive direction.

By comparing Figure 5b, Figure 6b, Figure 7b, Figure 8b and Figure 9b, it is found that the dynamic response curve of the moving electrode has three peaks. However, it is found that the middle peak is no longer obvious; this is because the vibration of the moving electrode within 1 ms is a forced vibration, and the moving electrode has been moving under the drive of inertial force. When the threshold of the switch reaches 350 g, the displacement of the movable electrode is about 50 microns. When the movable electrode recovers, the forced vibration has ended, so the peak value in the middle is not obvious.

**Figure 10:**
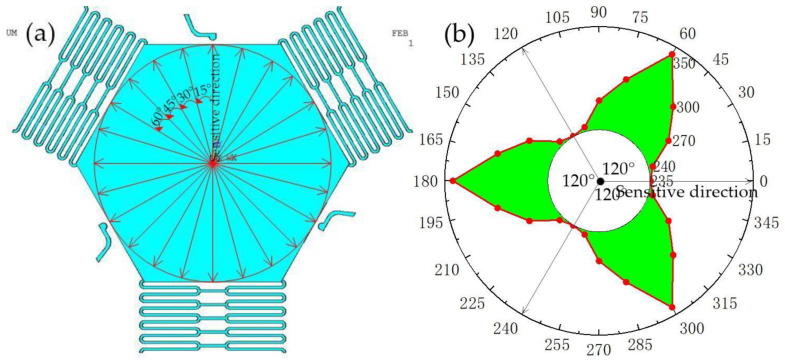
(**a**)Threshold distribution diagram of designed three-axis inertial switch., (**b**) the schematic diagram of the directions of applied accelerations.

The omnidirectional threshold analysis of the triaxial inertial switch was performed, as shown in Figure 10a. The analysis results show that the threshold distribution of the designed triaxial inertial switch is shown in Figure 10b, indicating that the designed triaxial inertial switch has the minimum threshold in the sensitive direction, which is 235 g. The farther it deviates from the sensitive direction, the greater the threshold. Therefore, the following conclusions can be drawn from the above simulation results: the designed triangular structure inertial switch can effectively improve the system stability when it receives the acceleration impact in the non-sensitive direction.

## 4. Fabrication and Test

### 4.1. Fabrication of the Prototype

**Figure 11:**
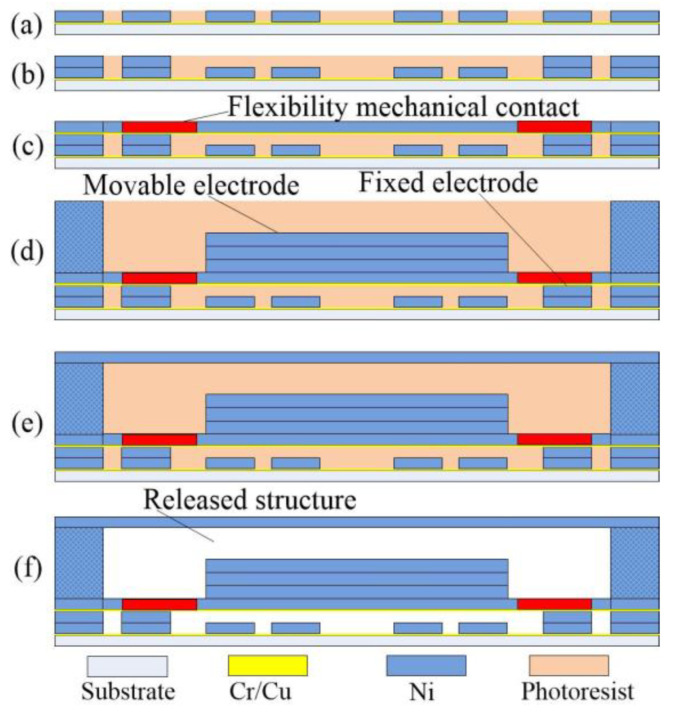
The diagram of fabrication process. (**a**) pad layer, (**b**) supporting layer, (**c**) spring layer, (**d**) proof mass, (**e**) anctilever beam, (**f**) released structure.

The three-axis inertial switch was fabricated by surface micromachining technology with nickel metal. The primary technology includes magnetron sputtering, etching, and electroplating, as shown in Figure 11.

(a) The chrome–copper conductive layer was prepared by magnetron sputtering. the bottom fixed electrode pattern was casted by photoresistance on the chrome–copper thin-film, and the Nickel-metal strips were prepared on the seed layer.

(b) A fixed support column was prepared on the bottom fixed electrode, and the protruding column can be used to suspend the movable electrode spring.

(c) Bottom layers of suspended spring and movable electrode were prepared by repeated magnetron film sputtering, photoresist spin coating, lithography, and electroplating techniques.

(d) The third layer of the chrome–copper film was fabricated and deposited, and the suspended fixed electrode was fabricated. The mass block was electroplated to a set thickness by repeated lithography and electroplating processes.

(e) The suspension interval was prepared above the mass block, and the upper cantilever was electroplated above the support structure.

(f) Finally, the photoresistance was removed, the movable structure of the device was released, and the chrome–copper film was selectively removed to obtain the mechanical inertial switch structure.

**Figure 12:**
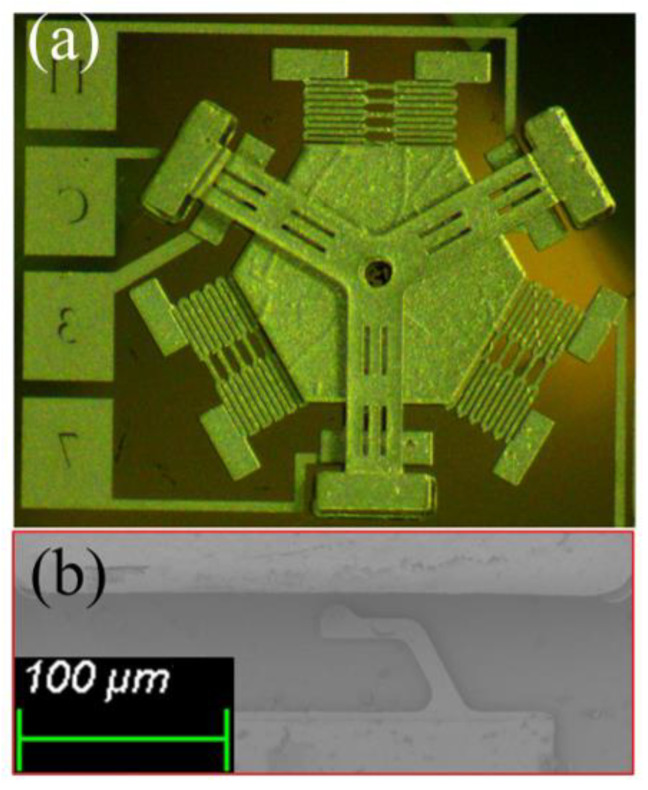
The photos of prototype. (**a**) the complete structure drawing of fabricated inertial switch, (**b**) the elastic fixed electrode.

The fabricated prototype was fabricated successfully, as shown in Figure 12a. The fixed electrode hidden under the cantilever beam is shown in Figure 12b.

### 4.2. Test of the Prototype

**Figure 13:**
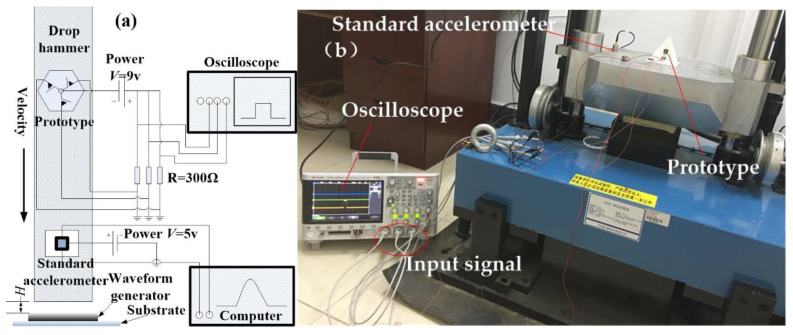
(**a**) The diagram of the dropping hammer system; (**b**) the photo of the test equipment.

The prototype was tested by a dropping hammer system (SuShi, SuZhou, China), and the test schematic diagram is shown in Figure 13a; the testing equipment is shown in Figure 13b. The prototype was fixed on the fixture, fabricated by 3D-printing technology. The prototype and standard acceleration were fixed on the objective table. One of the sensing directions of the device is perpendicular to the vibration plane. The electrodes in three sensitive directions were simultaneously connected with the signal input interface of the oscilloscope. When the acceleration reached the threshold value of the testing device, the inertial switch was activated, and the trigger signals were captured by the multi-channel oscilloscope.

**Figure 14:**
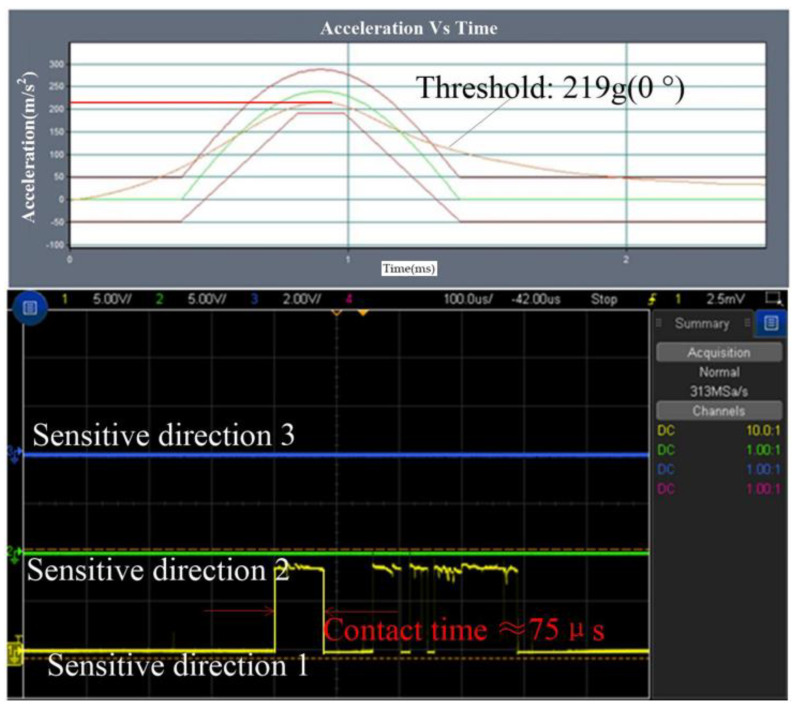
The tested threshold in one of the sensitive directions.

**Figure 15:**
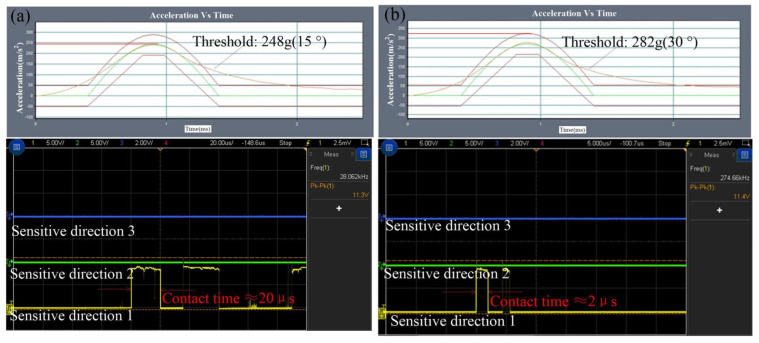

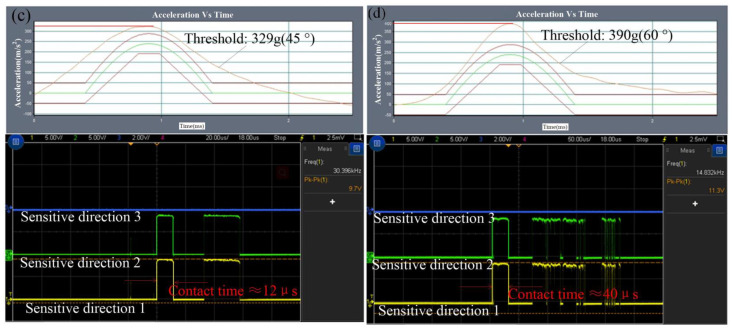
The tested results of thresholds in the non-sensitive directions. (**a**). acceleration curve and test signal when the impact acceleration deviates 15° from the sensitive direction; (**b**) acceleration curve and test signal when the impact acceleration deviates 30° from the sensitive direction; (**c**) acceleration curve and test signal when the impact acceleration deviates 45° from the sensitive direction; (**d**) acceleration curve and test signal when the impact acceleration deviates 60° from the sensitive direction.

The test result of the threshold in the sensitive direction is shown in Figure 14, which indicated that the threshold is about 219 g and the contact time is about 75 μs. The threshold is about 248 g in 15 degrees away from the sensitive direction, and the first contact time is about 20 μs, as shown in Figure 15a. The threshold is about 282 g, 329 g, and 390 g, and the contact time is about 2 μs, 12 μs and 40 μs when the applied acceleration is away from the sensitive direction by 30°, 45°, and 60°, respectively. The tested results are shown in Figure 15b–d, respectively.

The following conclusions can be drawn by testing the threshold values of the three-axis inertial switch in multiple directions:

1. When the applied acceleration’s direction deviates from the sensitive direction, the threshold increases with the increment of the angle, indicating that the designed three-axis sensitive inertial switch has the minimum threshold in the designed sensitive direction.
2. The contact time decreases first and then increases as the angle of acceleration deviates from the sensitive direction. Significantly, when the acceleration direction deviates from the sensitive direction by more than 45 degrees, the contact time increases when the active electrode contacts two fixed electrodes simultaneously under threshold acceleration. The analysis shows that the simultaneous action of the two electrodes hinders the elastic recovery of the movable electrode, thus prolonging the contact time.
3. The experimental results show that the threshold and contact time of the designed triaxial inertia switch is different from the simulation results. This is because there are errors between the experimental and design parameters, such as the line width of the suspension springs, the thickness of the mass block, the gap between the electrodes, and so on. At the same time, the test results also show that the larger the acceleration deviates from the sensitive direction, the larger the threshold, which was mutually verified with the simulation results.
4. The test results show that the contact time of the inertial switch in different directions is different from the simulation analysis, which is believed to be because the effect of friction resistance between electrodes is ignored in the simulation analysis, resulting in the difference between the theoretical analysis and the actual measured value.
5. When the acceleration is applied to the prototype in the direction away from the sensitive direction one of 60°, it is equivalent to the opposite direction of the sensitive direction three. The test results show that the fixed electrode contacted with the movable electrode in the sensitive direction one and two. However, the sensitive direction three does not contact all the time, indicating that the designed structure effectively improves the reverse anti-overload capability of the device.
6. To sum up, the three sensitive directions of the designed device do not interfere with each other, and the threshold difference between different directions is greater than 29 g. The dynamic stability of the designed inertial switch is verified.

## 5. Conclusions

In order to improve the dynamic stability of the inertial switch, a three-axis inertial switch with a triangular structure movable electrode is proposed. Static and simulation analysis methods analyzed the dynamic response performance. The static mechanism analysis results show that the proposed movable electrode structure can resist the acceleration impact from the non-sensitive direction, and the stability of the inertial switch response to the acceleration in the sensitive direction has been improved effectively. The simulation results show that the threshold value of the designed inertial switch is about 235 g in the sensitive direction. When the impact acceleration direction deviates from the sensitive direction by 15°, 30°, 45°, and 60°, The threshold for switching on is about 240 g, 270 g, 300 g, and 350 g, respectively. The results show that the triangular movable electrode structure can effectively resist the inertial impact of the non-sensitive direction and improve the stability of the suspension system. The experimental prototype was prepared by surface micromachining technology and tested by a dropping hammer system. The test results show that the designed inertia switch threshold is about 219 g, but the dynamic response characteristics of the prototype verify the rationality of the simulation analysis results. The error of contact time between tested results and simulation results is also analyzed. Finally, it is concluded that the dynamic stability of the vibration system can be improved by using the triangle-shaped structure as the movable electrode of the three-axis sensitive inertial switch.

## Figures and Tables

**Table 1:** The main structure parameters of three-axis inertial switch.

Structure	Movable Electrode				Fixed Beam		Gap			
**Parameters**	T	d	L	t_2_	t	t_1_	g	g_1_	H	h
**Values (μm)**	100	25	800	15	20	10	25	20	120	20

## Data Availability

Not applicable.
